# Supplementary material for: Patterns in the temporal complexity of global chlorophyll concentration
Source: Nat Commun. 2024 Feb 19;15:1522. doi: 10.1038/s41467-024-45976-8 (PMC10876569; doi:10.1038/s41467-024-45976-8)
Supplement: Supplementary file 1 — Supplementary Information [file 41467_2024_45976_MOESM1_ESM.pdf]

Supplementary material

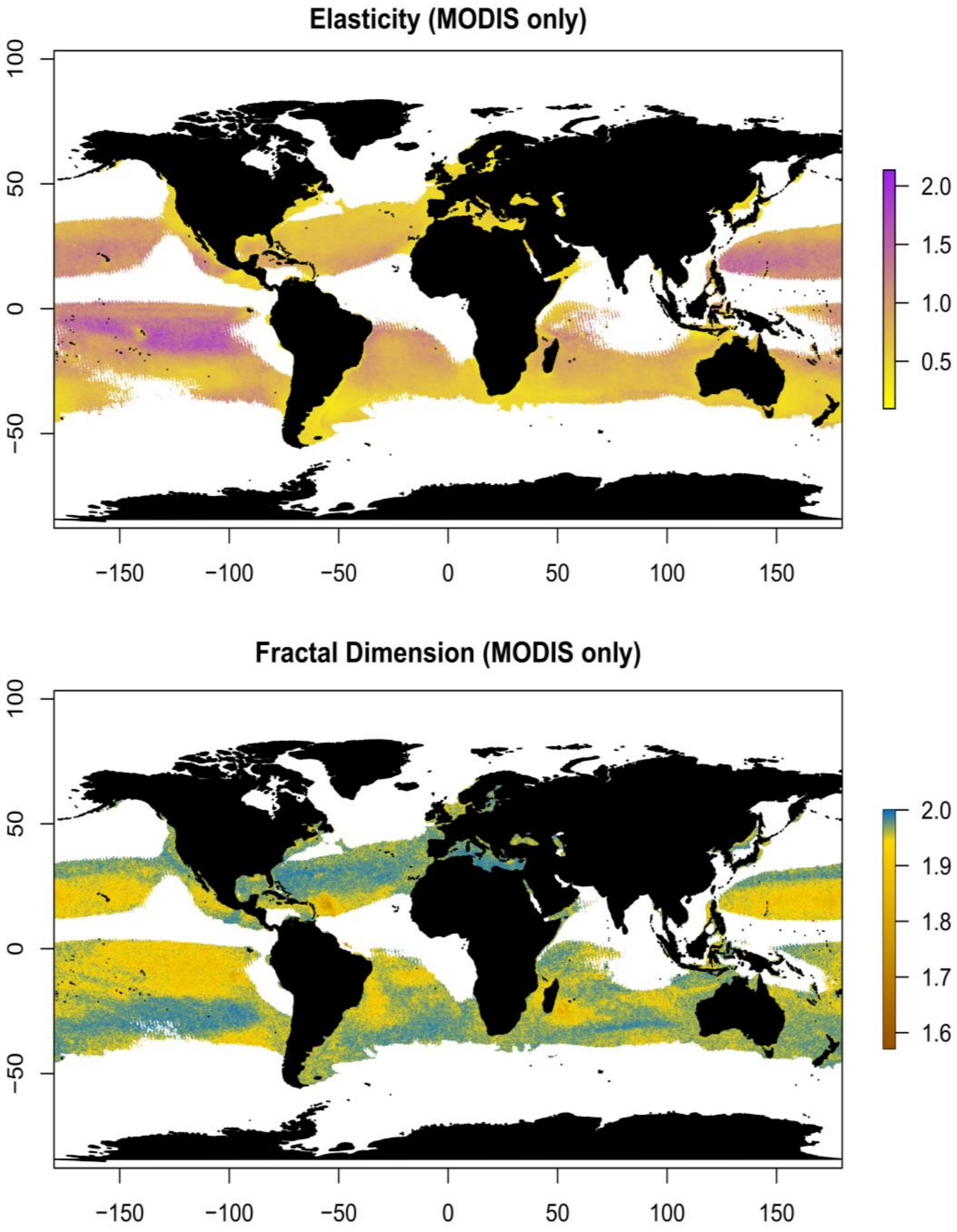

Figure S1: Global map of elasticity (top) and fractal dimension (bottom) calculated from 20-years (2002-2022) of daily-scale chlorophyll-*a* concentration time series as derived from MODIS observations alone. Darker colors (purple) indicate greater elasticity for the corresponding 25x25km pixel, whereas lighter colors (yellow) indicate lower elasticity. Similarly, blue, and yellow indicate a higher fractal dimension for the corresponding 25x25km pixel, whereas brown indicates lower fractal dimensions. Please note that the color scale for the map of fractal dimensions was chosen to highlight spatial differences. Areas in white are pixels where greater than 80% of the time series were missing observations or less than 400 days with consecutive measurements.

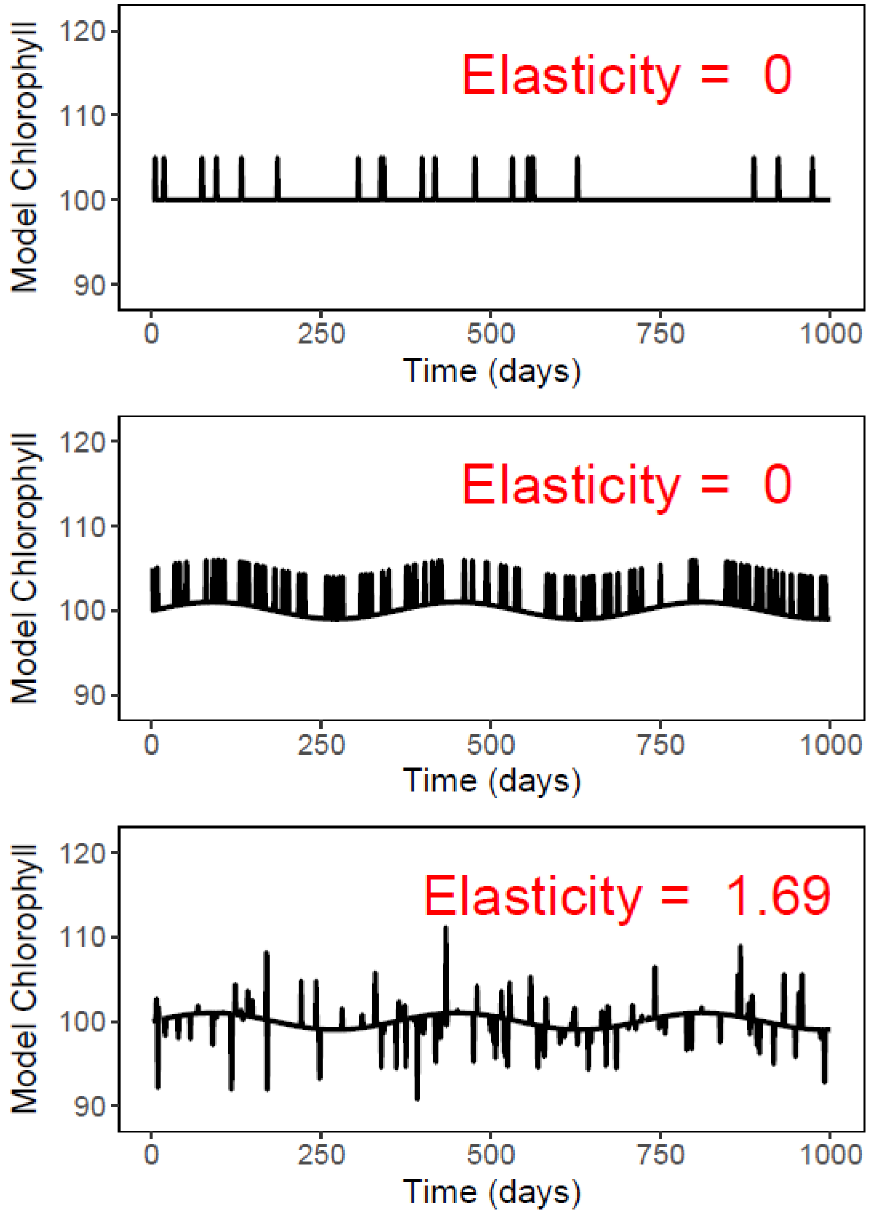

Figure S2: Model examples of varying elasticity in time series. Elasticity measures the sensitivity of a time series to threshold analysis, such that more variable day-to-day changes indicates greater complexity and results in a higher elasticity value.

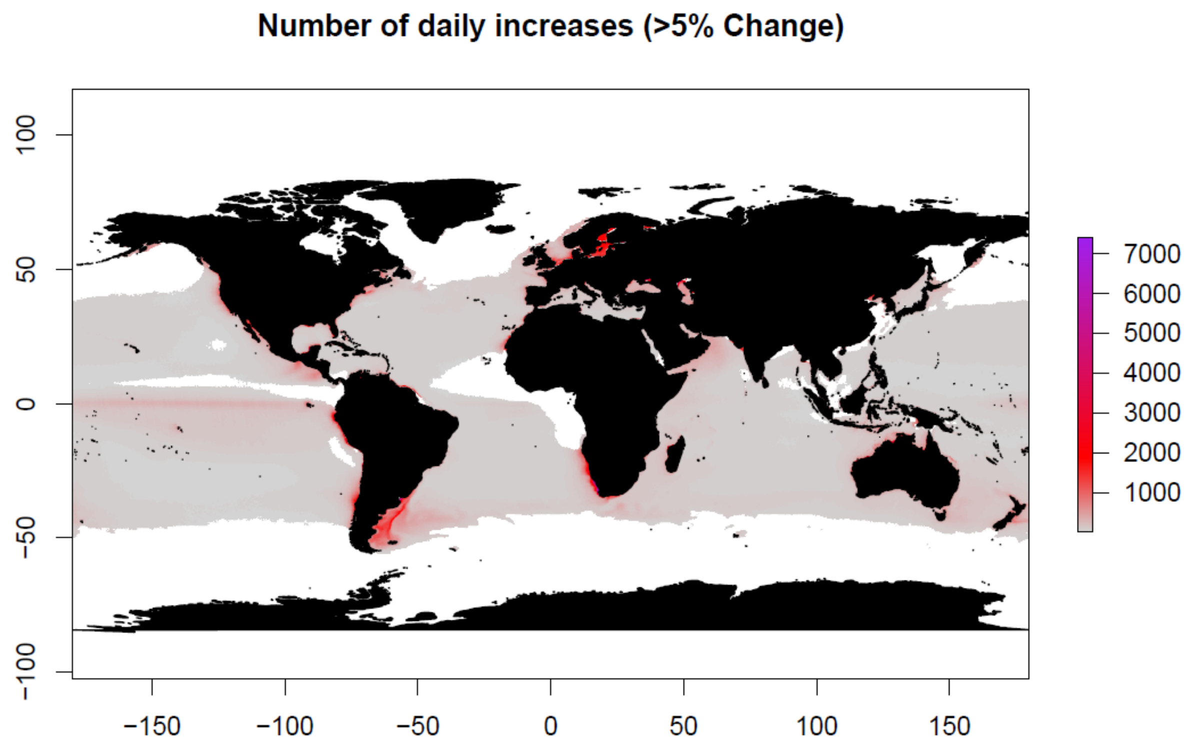

Figure S3: Number of days where the daily [chl-*a*] increase was greater than 5% of the median of the 25-year time series. Greater values (in red/purple) highlight the regions where the satellite observed more jumps in day-to-day [chl-*a*] (as estimated from the merged data product).

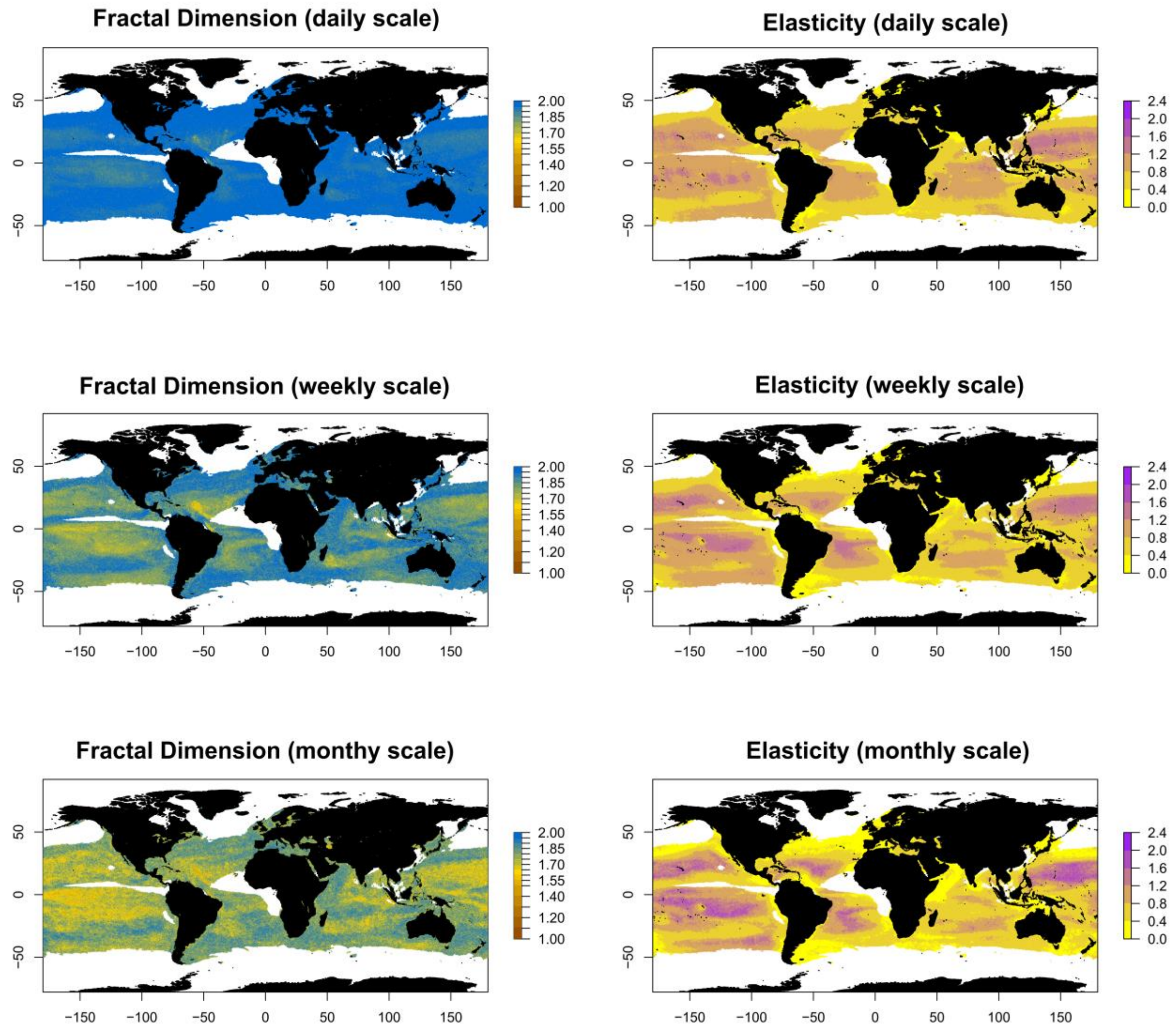

Figure S4: Global maps of the fractal dimension (left) and elasticity (right) for a range of temporal resolutions (top row – daily scale, middle row – weekly scale, bottom row – monthly scale). Darker colors (purple) indicate greater elasticity for the corresponding 25x25km pixel, whereas lighter colors (yellow) indicate lower elasticity. Similarly, blue, and yellow indicate a higher fractal dimension for the corresponding 25x25km pixel, whereas brown indicates lower fractal dimensions.

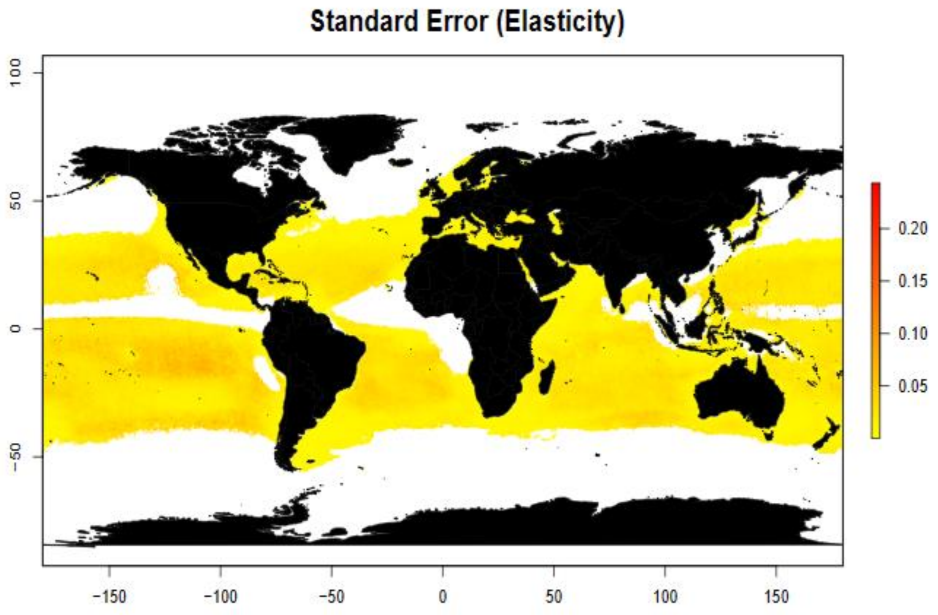

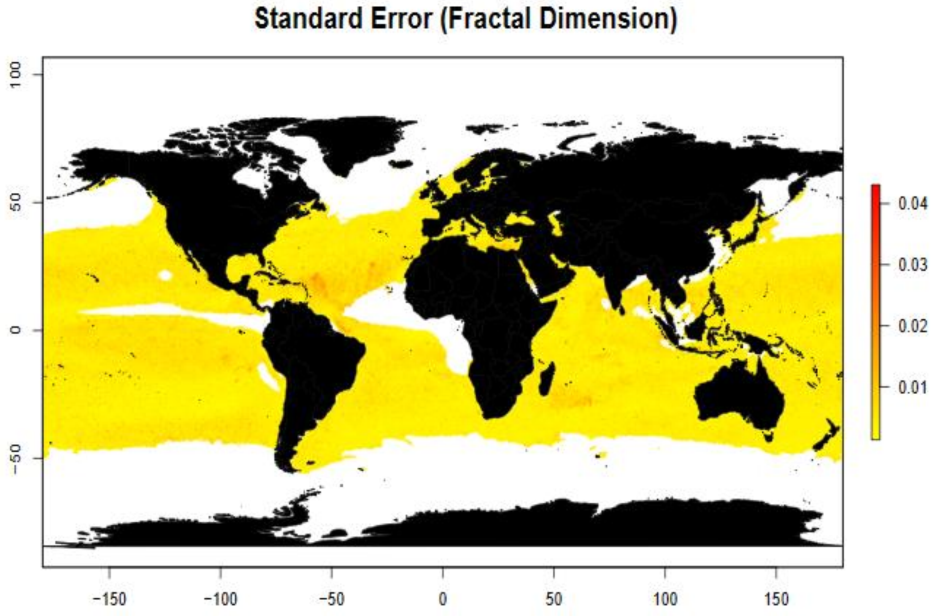

Figure S5: Global map of the standard error in elasticity (top) and fractal dimension (bottom) calculated from 25-years (1998-2022) of daily-scale chlorophyll-*a* concentration time series. Darker colors (red) indicate greater error for the corresponding 25x25km pixel, whereas lighter colors (yellow) indicate lower error. Areas in white are pixels where greater than 80% of the time series were missing observations or less than 400 days with consecutive measurements. Error was calculated from a distribution of elasticity and fractal dimension values for each pixel (30 randomly selected temporal windows of 4000-days each).

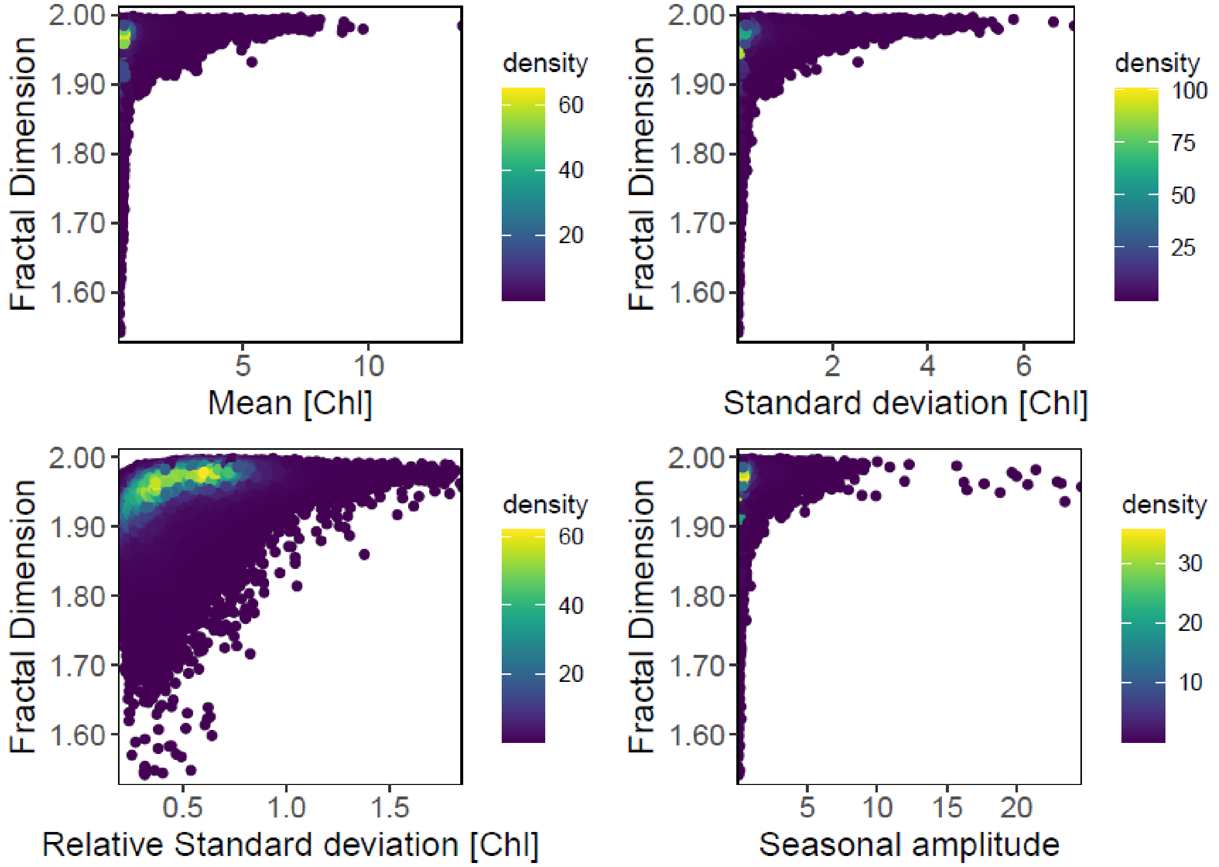

Figure S6: Relationship between the fractal dimension of each time series and the mean chlorophyll-*a* concentration (top left), the standard deviation (top right), the relative standard deviation (bottom left) and the seasonal amplitude (bottom right). Seasonal amplitude was calculated by subtracting the highest monthly average chlorophyll-*a* concentration from the lowest monthly average chlorophyll-*a* concentration. Density was estimated using Two-Dimensional Kernel Density Estimation ('kde2d' function in the 'MASS' package in R). There are 362,969 points in each plot.

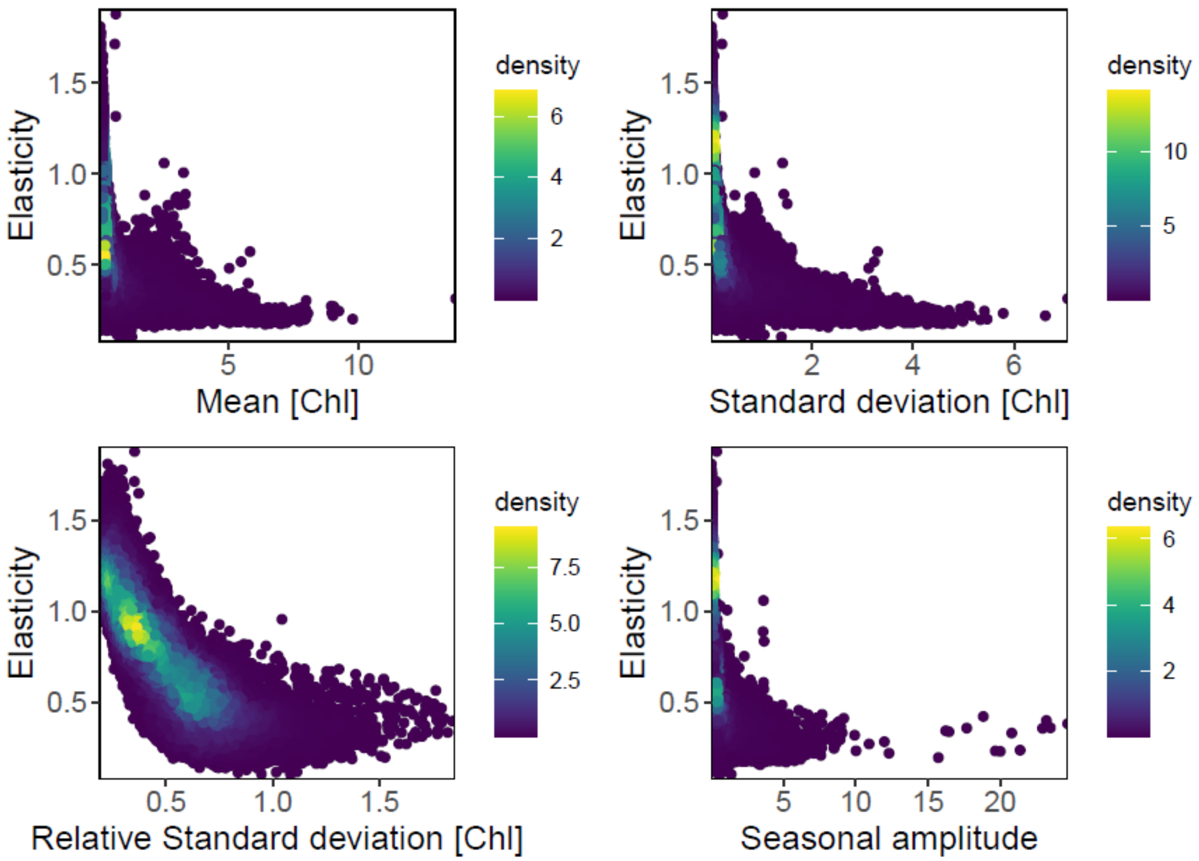

Figure S7: Relationship between the elasticity of each time series and the mean chlorophyll-*a* concentration (top left), the standard deviation (top right), the relative standard deviation (bottom left) and the seasonal amplitude (bottom right). Seasonal amplitude was calculated by subtracting the highest monthly average chlorophyll-*a* concentration from the lowest monthly average chlorophyll-*a* concentration. Density was estimated using Two-Dimensional Kernel Density Estimation ('kde2d' function in the 'MASS' package in R). There are 362,969 points in each plot.

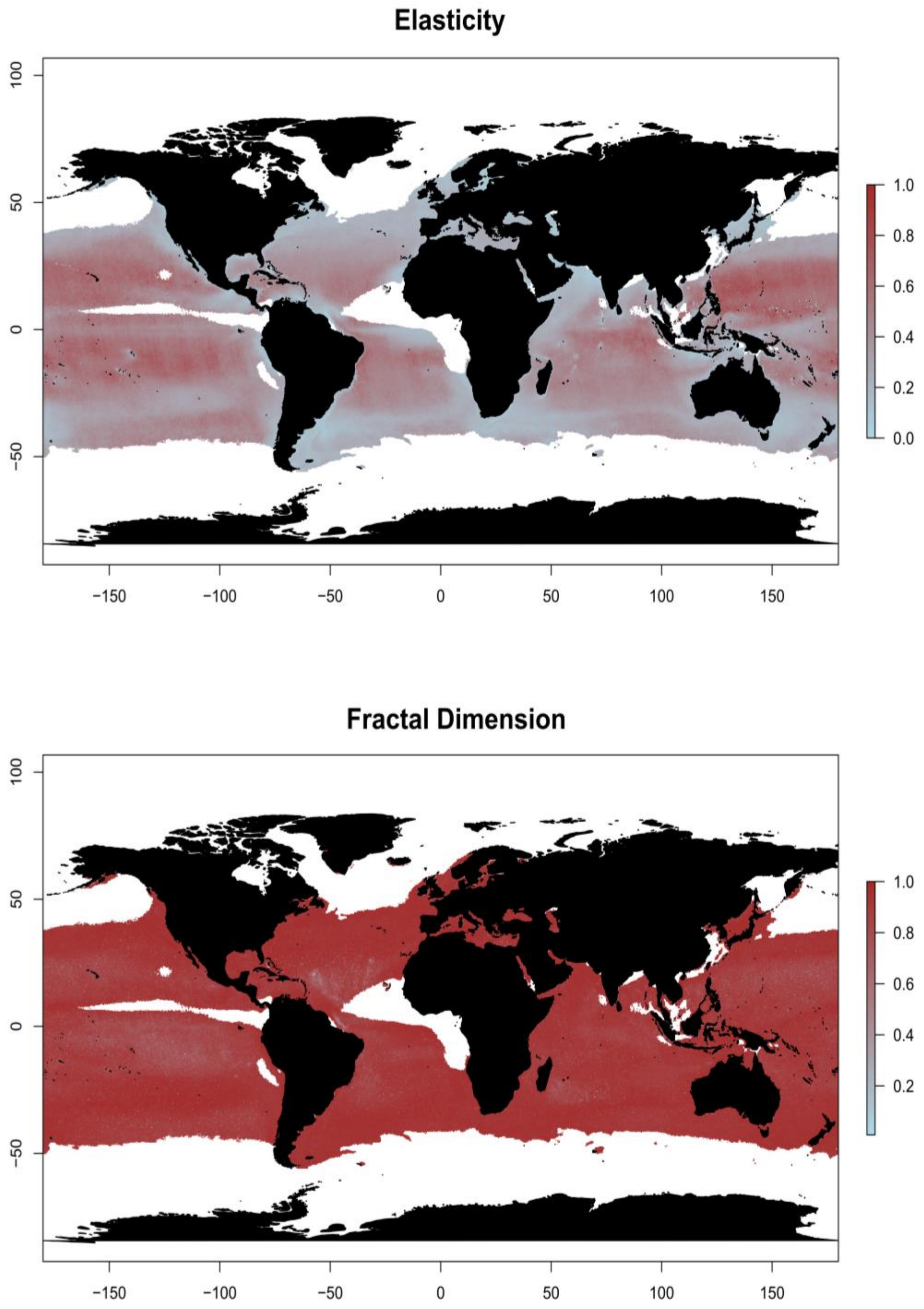

Figure S8: Global map of elasticity (top) and fractal dimension (bottom) calculated from 25-years (2002-2022) of daily-scale chlorophyll-*a* concentration time series. Darker colors (red) indicate greater elasticity and fractal dimension for the corresponding 25x25km pixel, whereas lighter colors (grey) indicate lower elasticity and fractal dimension. Areas in white are pixels where greater than 80% of the time series were missing observations or less than 400 days with consecutive measurements. Both elasticity and fractal dimension have been min-max normalized and plotted on the same color scale.

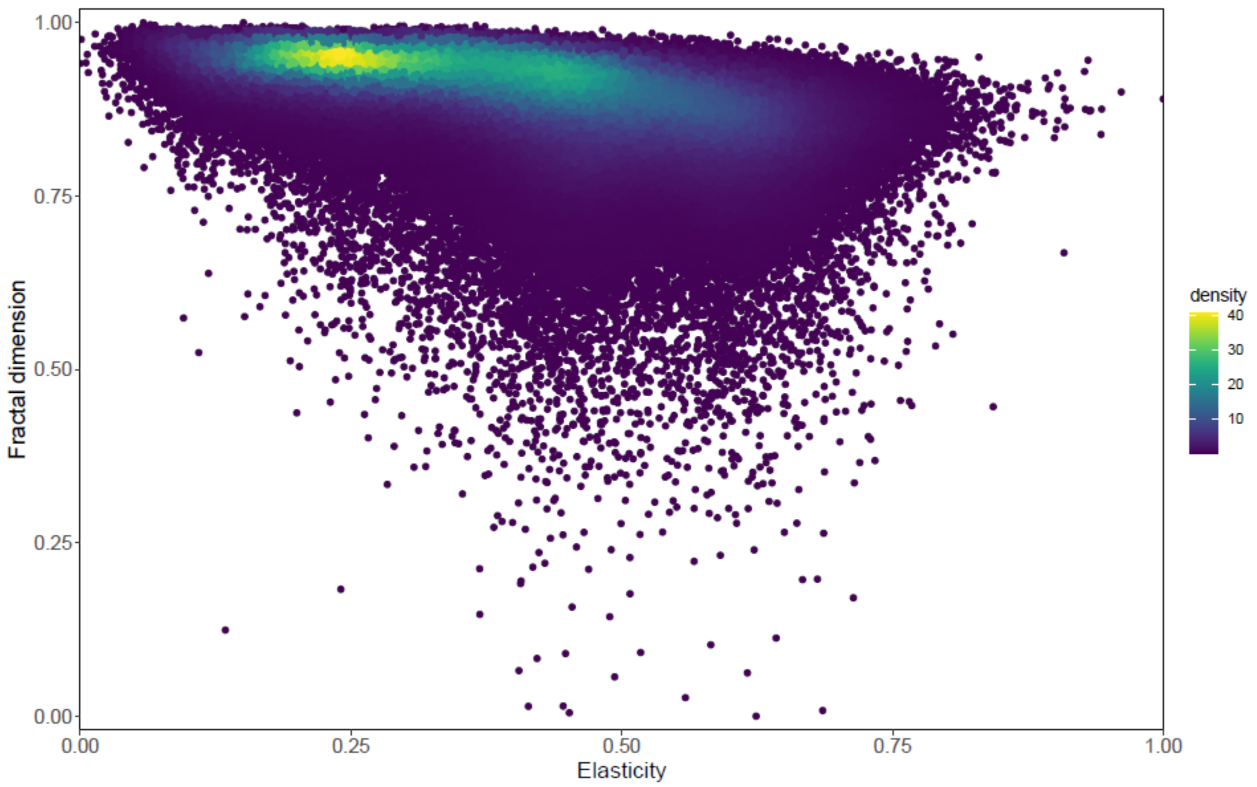

Figure S9: Relationship between the fractal dimension and elasticity of each chl-*a* concentration time series. Density was estimated using Two-Dimensional Kernel Density Estimation ('kde2d' function in the 'MASS' package in R). There are 362,969 points in this plot.

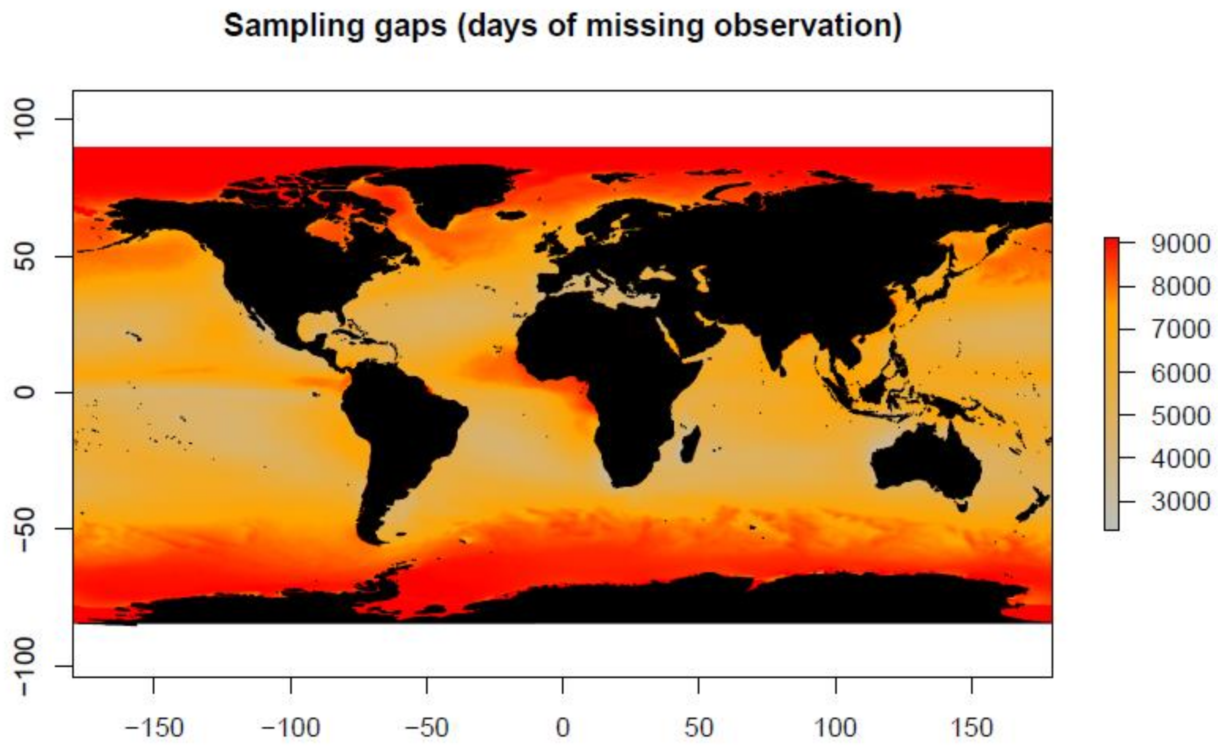

Figure S10: Global map of sampling gaps from 25-years (1998-2022) of [chl-*a*] observations on a 25km scale. Darker red colors indicate more days of missing samples due to insufficient satellite coverage or quality-control issues. For most of the analyses, a cut-off of ~80% (7300 days) was used to remove pixels that did not have enough data to form a time series of [chl-*a*].

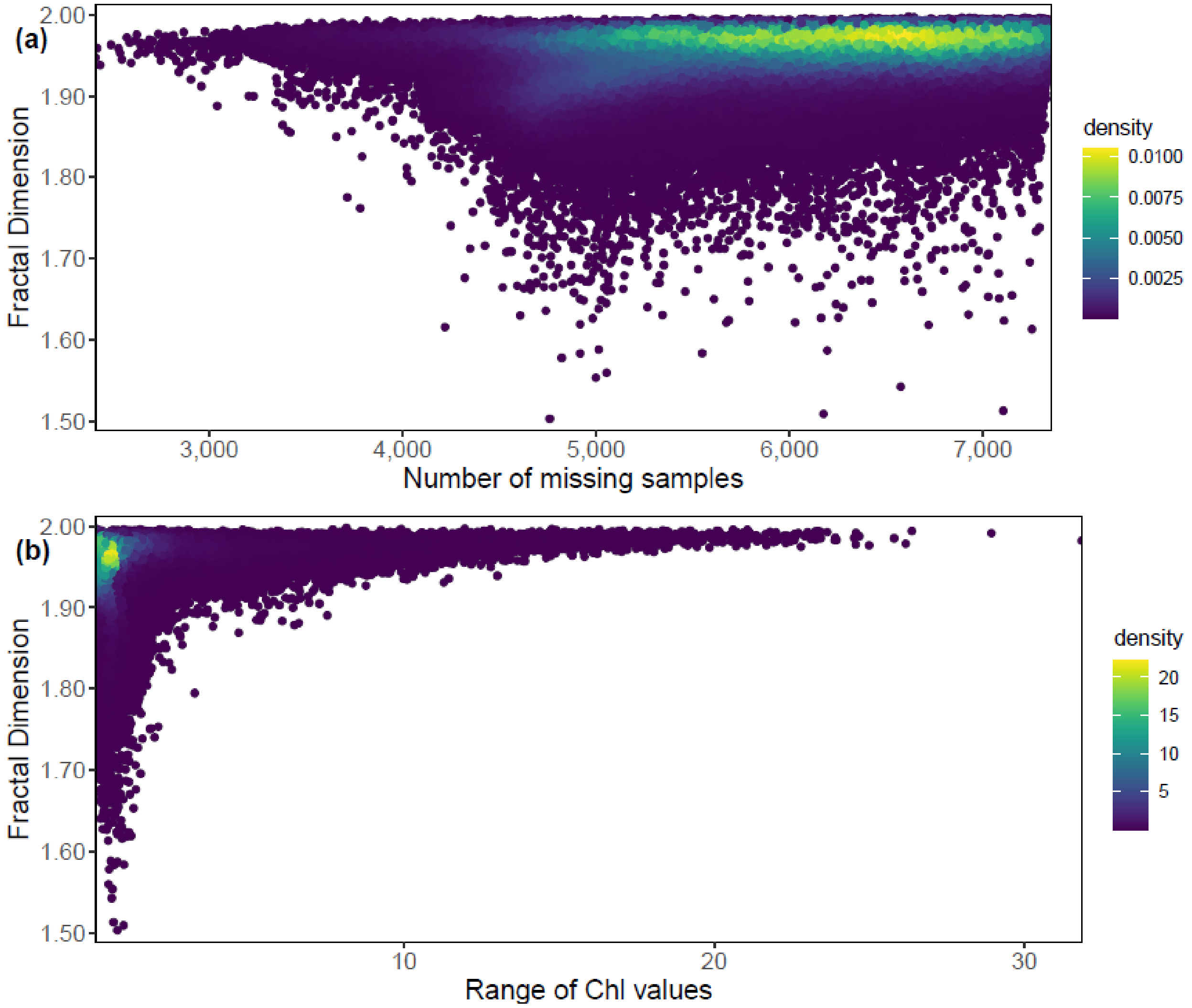

Figure S11: Relationship between the fractal dimension of each time series with (a) the number of missing daily observations that were randomly resampled and (b) the range (maximum value – minimum value) of the time series. Neither factor appears to have a strong relationship to the calculated fractal dimension. Density was estimated using Two-Dimensional Kernel Density Estimation ('kde2d' function in the 'MASS' package in R). There are 362,969 points in each plot.

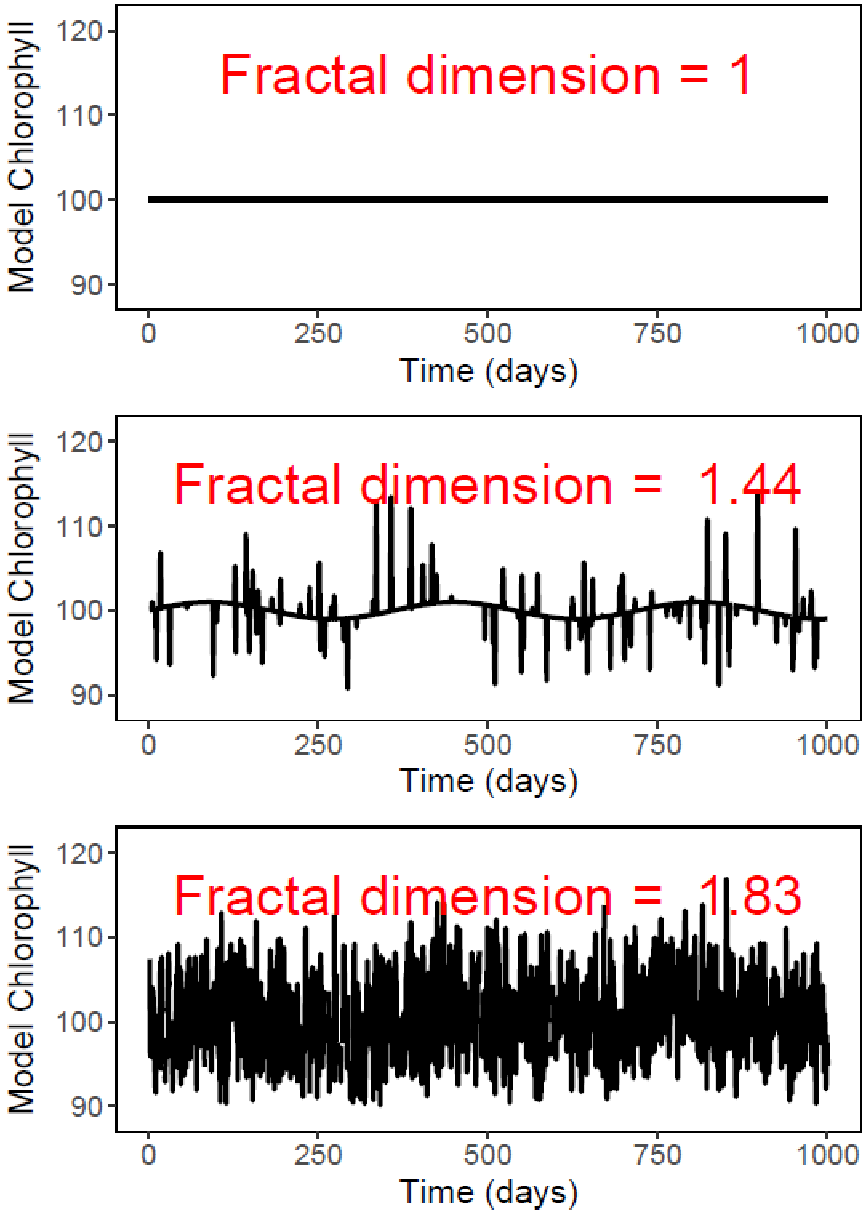

Figure S12: Model examples of varying fractal dimension in time series. A smooth time series has a fractal dimension of 1. Increasing the "roughness" (incorporating long tails or large deviations) results in a higher fractal dimension (1-2).

Supplementary Table 1: Sample sizes for Figure 3a,b and c.

Variable	**Year**	**Sample size (25x25km pixels)**
Mean chlorophyll-*a* concentration ($mg\ m^{-3}$)	1998	85,238
	1999	101,481
	2000	89,781
	2001	83,273
	2002	243,733
	2003	332,829
	2004	338,032
	2005	334,064
	2006	330,365
	2007	329,903
	2008	296,993
	2009	298,798
	2010	310,552
	2011	251,650
	2012	270,605
	2013	254,576
	2014	256,867
	2015	258,885
	2016	260,290
	2017	255,915
	2018	256,898
	2019	259,997
	2020	251,039
	2021	253,674
	2022	318,138
Elasticity	1998	57
	1999	34
	2000	45
	2001	15
	2002	6,755
	2003	90,812
	2004	89,945
	2005	88,153
	2006	90,048
	2007	84,085
	2008	44,095
	2009	34,747
	2010	45,484
	2011	6,316
	2012	38,804
	2013	26,481
	2014	27,135
	2015	27,457
	2016	26,418
	2017	28,623
	2018	29,817

	2019	32,138
	2020	22,741
	2021	22,099
	2022	94,155
Fractal dimension	1998	85,238
	1999	101,481
	2000	89,781
	2001	83,273
	2002	243,733
	2003	332,829
	2004	338,032
	2005	334,064
	2006	330,365
	2007	329,903
	2008	296,993
	2009	298,798
	2010	310,552
	2011	251,650
	2012	270,605
	2013	254,576
	2014	256,867
	2015	258,885
	2016	260,290
	2017	255,915
	2018	256,898
	2019	259,997
	2020	251,039
	2021	253,674
	2022	318,138
