# Supplementary material for: Patterns in the temporal complexity of global chlorophyll concentration
Source: Nat Commun. 2024 Feb 19;15:1522. doi: 10.1038/s41467-024-45976-8 (PMC10876569; doi:10.1038/s41467-024-45976-8)
Supplement: Supplementary file 3 — Reporting Summary [file 41467_2024_45976_MOESM3_ESM.pdf]

nature portfolio

Corresponding author(s): Vitul Agarwal

Last updated by author(s): Jan 15, 2024

Reporting Summary

Nature Portfolio wishes to improve the reproducibility of the work that we publish. This form provides structure for consistency and transparency in reporting. For further information on Nature Portfolio policies, see our Editorial Policies and the Editorial Policy Checklist.

Statistics

For all statistical analyses, confirm that the following items are present in the figure legend, table legend, main text, or Methods section.

n/a	Confirmed	
☐	☒	The exact sample size (*n*) for each experimental group/condition, given as a discrete number and unit of measurement
☒	☐	A statement on whether measurements were taken from distinct samples or whether the same sample was measured repeatedly
☒	☐	The statistical test(s) used AND whether they are one- or two-sided<br>*Only common tests should be described solely by name; describe more complex techniques in the Methods section.*
☒	☐	A description of all covariates tested
☐	☒	A description of any assumptions or corrections, such as tests of normality and adjustment for multiple comparisons
☐	☒	A full description of the statistical parameters including central tendency (e.g. means) or other basic estimates (e.g. regression coefficient) AND variation (e.g. standard deviation) or associated estimates of uncertainty (e.g. confidence intervals)
☒	☐	For null hypothesis testing, the test statistic (e.g. F, t, r) with confidence intervals, effect sizes, degrees of freedom and P value noted<br>*Give P values as exact values whenever suitable.*
☒	☐	For Bayesian analysis, information on the choice of priors and Markov chain Monte Carlo settings
☒	☐	For hierarchical and complex designs, identification of the appropriate level for tests and full reporting of outcomes
☒	☐	Estimates of effect sizes (e.g. Cohen's *d*, Pearson's *r*), indicating how they were calculated

Our web collection on statistics for biologists contains articles on many of the points above.

Software and code

Policy information about availability of computer code

Data collection	No software was used for data collection.
Data analysis	All the analyses and plotting were conducted in R using the R packages “raster”, “maps”, “ggplot2”, “cowplot” , "fractaldim", "MASS", "viridis” and “ncdf4”. The code required for the analysis is available at https://github.com/vitul-agarwal-1/chl-complexity (https://doi.org/10.5281/zenodo.10498357).

For manuscripts utilizing custom algorithms or software that are central to the research but not yet described in published literature, software must be made available to editors and reviewers. We strongly encourage code deposition in a community repository (e.g. GitHub). See the Nature Portfolio guidelines for submitting code & software for further information.

Data

Policy information about availability of data

All manuscripts must include a data availability statement. This statement should provide the following information, where applicable:

- Accession codes, unique identifiers, or web links for publicly available datasets
- A description of any restrictions on data availability
- For clinical datasets or third party data, please ensure that the statement adheres to our policy

Remote sensing data are available from https://hermes.acri.fr/. Data can be downloaded for specific locations and time periods under “GlobColour data search”. Source data for each of the figures are provided with this paper. Sample datasets (processed) to reproduce part of the analysis can be found at https://doi.org/10.5281/zenodo.10498362

Research involving human participants, their data, or biological material

Policy information about studies with human participants or human data. See also policy information about sex, gender (identity/presentation), and sexual orientation and race, ethnicity and racism.

Reporting on sex and gender	n/a
Reporting on race, ethnicity, or other socially relevant groupings	n/a
Population characteristics	n/a
Recruitment	n/a
Ethics oversight	n/a

Note that full information on the approval of the study protocol must also be provided in the manuscript.

Field-specific reporting

Please select the one below that is the best fit for your research. If you are not sure, read the appropriate sections before making your selection.

☐ Life sciences ☐ Behavioural & social sciences ☒ Ecological, evolutionary & environmental sciences

For a reference copy of the document with all sections, see nature.com/documents/nr-reporting-summary-flat.pdf

Ecological, evolutionary & environmental sciences study design

All studies must disclose on these points even when the disclosure is negative.

Study description	This study utilized publicly available chlorophyll-a concentration data to develop mathematical metrics and quantify the complexity of global ocean chlorophyll time series. Each 25km by 25km pixel was considered unique and calculations were performed independently for each time series. There were no replicates of pixels. There were 362,969 pixels that had sufficient data for analysis.
Research sample	The data were a merged satellite product that combined observations from from the Sea-viewing Wide Field of View Sensor (SeaWiFS), Moderate Resolution Imaging Spectroradiometer on the Aqua satellite (MODIS), Medium Resolution Imaging Spectrometer (MERIS), Visible and Infrared Imaging/Radiometer Suite (VIIRS) and the Ocean and Land Color Instruments (OLCI-A and OLCI-B) sensors. A merged product was used to increase data availability on a daily scale.
Sampling strategy	No sample size calculations were performed. All pixels with sufficient data were considered to be part of the global analysis.
Data collection	All the satellite data are publicly available and were downloaded from existing repositories (GlobColour).
Timing and spatial scale	The spatial scale used in the study was 25km. The time series data (Jan 1998 - Jan 2023) were evaluated on a daily scale.
Data exclusions	There were several steps that removed or filtered data. All global locations where less than 20% of days of had chlorophyll data were excluded from further analysis (i.e. many high latitude regions). Only locations that had greater than 400 days of consecutive observations were considered in the calculations measuring elasticity. For the calculation of fractal dimensions, any daily measurements that exceeded 3 standard deviations from the mean were removed as erroneous outliers. When calculating yearly change, only locations where there were at least 100 days of consecutive samples were considered in the analysis.
Reproducibility	The analysis is reproducible using the available satellite data and applying the calculations as indicated in the paper. This study did not involve lab experiments. The calculations were performed multiple times with only minor differences between iterations depending on the parameter values.

Randomization	Randomization was not possible for this study. Time series were not allocated into groups.
Blinding	Blinding is not relevant to this study. As the study relied on satellite data, there were no participants except the authors.

Did the study involve field work? ☐ Yes ☒ No

Reporting for specific materials, systems and methods

We require information from authors about some types of materials, experimental systems and methods used in many studies. Here, indicate whether each material, system or method listed is relevant to your study. If you are not sure if a list item applies to your research, read the appropriate section before selecting a response.

Materials & experimental systems

n/a	Involved in the study	
☒	☐	Antibodies
☒	☐	Eukaryotic cell lines
☒	☐	Palaeontology and archaeology
☒	☐	Animals and other organisms
☒	☐	Clinical data
☒	☐	Dual use research of concern
☒	☐	Plants

Methods

n/a	Involved in the study	
☒	☐	ChIP-seq
☒	☐	Flow cytometry
☒	☐	MRI-based neuroimaging

Plants

Seed stocks	*Report on the source of all seed stocks or other plant material used. If applicable, state the seed stock centre and catalogue number. If plant specimens were collected from the field, describe the collection location, date and sampling procedures.*
Novel plant genotypes	*Describe the methods by which all novel plant genotypes were produced. This includes those generated by transgenic approaches, gene editing, chemical/radiation-based mutagenesis and hybridization. For transgenic lines, describe the transformation method, the number of independent lines analyzed and the generation upon which experiments were performed. For gene-edited lines, describe the editor used, the endogenous sequence targeted for editing, the targeting guide RNA sequence (if applicable) and how the editor was applied.*
Authentication	*Describe any authentication procedures for each seed stock used or novel genotype generated. Describe any experiments used to assess the effect of a mutation and, where applicable, how potential secondary effects (e.g. second site T-DNA insertions, mosiacism, off-target gene editing) were examined.*
